# Core competencies for health headquarters: a systematic review and meta-synthesis

**DOI:** 10.1186/s12889-020-08884-2

**Published:** 2020-06-09

**Authors:** Hamed Fattahi, Hasan Abolghasem Gorji, Mahboubeh Bayat

**Affiliations:** 1 School of Health Management and Information Sciences, Iran University of Medical Sciences, Tehran, Iran; 2 Health Management and Economics Research Center, Iran University of Medical Sciences, Tehran, Iran; 3 Gerash University of Medical Sciences, Gerash, Iran; 4 Center for Health Human Resources Research & Studies, Ministry of Health and Medical Education, Tehran, Iran

**Keywords:** Competency, Headquarter, Health sector, Workforce

## Abstract

**Background:**

The availability of human resources for the health sector is not enough requirement for addressing health needs. Instead, it is necessary to take effective steps to meet the requirements of the health care system in case the system has the necessary competencies. This study was performed to identify the competencies of health headquarters in meeting the needs of the health system.

**Methods:**

This thematic synthesis was performed to develop a set of central themes that summarize all the topics raised in the articles reviewed in this study. The quality of the articles was assessed by the Standards for Reporting Qualitative Research.

**Results:**

We included 12 articles from seven countries. Seven central themes were inductively developed from the analysis: (1) Leadership and management, (2) Analyzing, interpreting, and reporting, (3) Public health knowledge, (4) Interpersonal relationship, (5) Personality competencies, (6) Cultural and community competencies, and (7) International/Global health competencies.

**Conclusion:**

The findings of this review may help to address how to recruit and retain health headquarters, optimize the headquarters ability and expertise, and develop some approaches to promote their scientific, practical, and professional levels. These issues can drive the organization toward their visions, strategies, and great objectives.

## Background

Today, successful managers believe that the employees are valuable assets providing the competitive benefits that must keep the organizations in the competition arena. Organizations are now aware of the potential and emerging perspective of human resources strategy and the central and incrementing role of intangible assets and intellectual capital [1–3].

Many countries in the world face critical challenges regarding human resources, such as lack of human resources, lack of a balance in skills combination, and failure in knowledge and skills of health staff. In this regard, the workforce is the heart of the health system based on which the health and survival of the health system are established [4, 5].

Nevertheless, human factors are ignored in most cases in macro planning. Considering the human factors and their functions is a fundamental prerequisite in achieving organizational goals and enable HR managers to take the necessary steps at the right time to ensure satisfying the objectives [6, 7].

Under these conditions, health systems are required to pay attention to the basic competencies of their human resources. This includes policymaking, planning, management, and governance, supported by evidence from the analysis of accurate and complete data for the health workforce. An effective workforce in the health field does not merely mean employing a sufficient number of these forces; rather, it includes associated behaviors or activities, types of knowledge, skills, and motivations as the behavioral, technical, and motivational prerequisites for successful performance in a given role or job [8].

Today, all countries at every level of social-economic development encounter specific problems in the education, provision, maintenance, and functioning of their human resources [9, 10]. In this condition paying attention to competencies of health headquarters that work at the health official government organizations or the top of a national level taking full responsibility for managing all health activity is crucial for any country [11].

A competency approach is based on the concept of the essential characteristics of individuals leading the efficient and excellent performance within a job or task. This approach has created a new space in the human resource development horizon. Within this framework, the main role of competencies is to integrate the core components into human resource development in organizations [12, 13].

Therefore, the issue of competences has been addressed in the civil service laws of countries such as the United States, Canada, the United Kingdom, Germany, Sweden, and Japan [14, 15].

Reflection on how to ensure that the Health headquarters who are being employed have the necessary competencies for value creation in the organization reveals the importance and necessity of developing human and social capitals, particularly in organizations with sophisticated products and high technology [16]. In this regard, determining competencies is the first step to identify training needs and to implement management development programs [17].

Given the pre-stated issues and the importance of competency-based HR planning, the most fundamental task for establishing competency-based HR management in government agencies is competency-based HR planning [18] The significant role of human resource managers in the success of organizations, especially health care organizations, and the role of health headquarters in promoting the overall goals of the health system based on the horizon of development plans of any country, can be considered among the most important priorities in the field of human resources in the health sector. Therefore, this study was performed to identify the competencies of health headquarters.

## Methods

### Design

Our systematic meta-synthesis review complies with the PRISMA guidelines [19] to explain the above-mentioned purposes. Also, we employed the method proposed by Thomas & Harden [20] to analyze data. This procedure involves three steps: 1) the coding of text, 2) the development of descriptive themes, and 3) producing analytical themes. In this procedure, we included and consulted members of a formal advisory group to integrate different perspectives, improve the literature, and to guarantee the applicability of our review results.

### Search strategy

This systematic meta-synthesis review was conducted in December 2019, using four electronic databases of Web of Science, Scopus, PubMed, and Embase. Targeted search strategies were initially developed in consultation with our team’s librarian. Despite numerous related but not on-topic literature, there is no proper study in this regard. Thus, the search strategy was concentrated on identifying the key terms in titles to more proficiently target findings using the keywords: TITLE (“Competenc*” OR “Capabilities” OR “Abilities” OR “Skills”) AND (“Headquarters” OR “Staff” OR “Health worker*” OR “Employees” OR “personnel” OR “Support worker”) AND (“Healthcare organizations” OR “Health organization*” OR “Health sector” OR “Public health organization*” OR “Public health sector”).

Our search sources and strategies involved a) health, healthcare, and interdisciplinary electronic databases; b) grey literature sources; c) manual searching for related specialized key journals; and d) reference lists in publications recognized in (a), (b), and (c). All types of documents were searched focusing on qualitative research studies. Search limits were applied in publication date (between 1980 and 2019). Search results were imported into an Endnote© version 7.

### Eligibility criteria

For Inclusion criteria in this review study, all papers with a (i) qualitative method (Ethnography, Narrative, Phenomenological Grounded Theory, mixed method) (ii) published between 1980 and 2019 were considered. The reason for selecting this time range is that competency management was first introduced during the 1980s [21]. (iii) All studies concerning the health headquarters competencies were included. Because of the small number of articles available on the competencies of health headquarters, the research team attempted to use articles available and the level of competencies that is necessary for health headquarters. Participants were selected among those who are experts in health area fields. The exclusion criteria resulted in the non-inclusion of studies (i) using quantitative methods and (ii) not reported on the competencies in headquarters and (iii) not related to health sectors were excluded. Also, there was no exclusion based on language.

### Study selection

Two reviewers originally reviewed the headings and certain articles discussing the competencies of health workers for the abstract review. All listed headings were directly inserted into Endnote© V.7 (Thomson Reuters, Philadelphia, PA, USA). Then, duplicate data were eliminated. Through the abstract review step, all works on the health workforce and competencies related to the health sector not coping with health headquarters (health headquarters that work at the health official government organizations or the top of a national level) were excluded. Followed by limiting abstracts to the associated health headquarters in terms of competencies, a complete text review was performed to classify the studies coping with health headquarters competencies. Here, a full analysis of all those papers was performed, and particular articles were identified stating global health competencies within the health headquarters. Investigating the bibliographies of these papers, a cross-search was conducted to recognize any further related articles.

### Quality appraisal

To deliberate the results and conclusions of the studies and judge the integrity and value of the utilized data, investigating the articles’ quality was a crucial phase of this procedure. The quality of the qualitative papers was evaluated using the Standards for Reporting Qualitative Research (SRQR) [22] guidelines for qualitative studies.

This valuation was performed by three authors independently and then the outcomes were discussed within the research group until achieving the agreement. Due to the lack of consensus regarding the function and role of study quality evaluation as part of systematic reviews [23], no study was excluded from the analysis in our review. Nevertheless, a limited number of quality studies seemed to play an insignificant role in synthesis [24].

### Data extraction

The applicable results were extracted and tabulated by re-reading the chosen article including features of each study, participants’ demographics, and details of any interesting results. Study selection and data extraction were carried out and examined by authors over data extraction. Each article was critically evaluated in terms of methodological coherence utilizing criteria defined in the (Standards for Reporting Qualitative Research).

### Qualitative synthesis

Our analysis followed the procedure described by Thomas and Harden [20]. Our analysis initiated with a concentrated reading and then re-readings of each article’s headings, abstracts, and texts. The formal features of the studies were extracted by one researcher. Then, three researchers independently extracted and analyzed the first-order data (the study results) and the second-order results (authors’ discussions and interpretations of the findings) of each study selected. The independent analyses were then compared and deliberated at the research meetings. The data were managed and the themes were developed using MAXQDA qualitative analysis software. By thematic analysis, it was made possible to develop themes inductively from the study data. The translation phase included comparison and assembling the themes attained by analyzing each paper to preserve the key themes capturing similar ideas in the various articles and then to develop overarching ideas regarding the research question. Triangulating both the data sources and the analyses was performed to obtain the high rigor level of the data including three independent analyses and weekly research meetings to deliberate the findings [25].

## Results

### Study selection

**Fig. 1:**
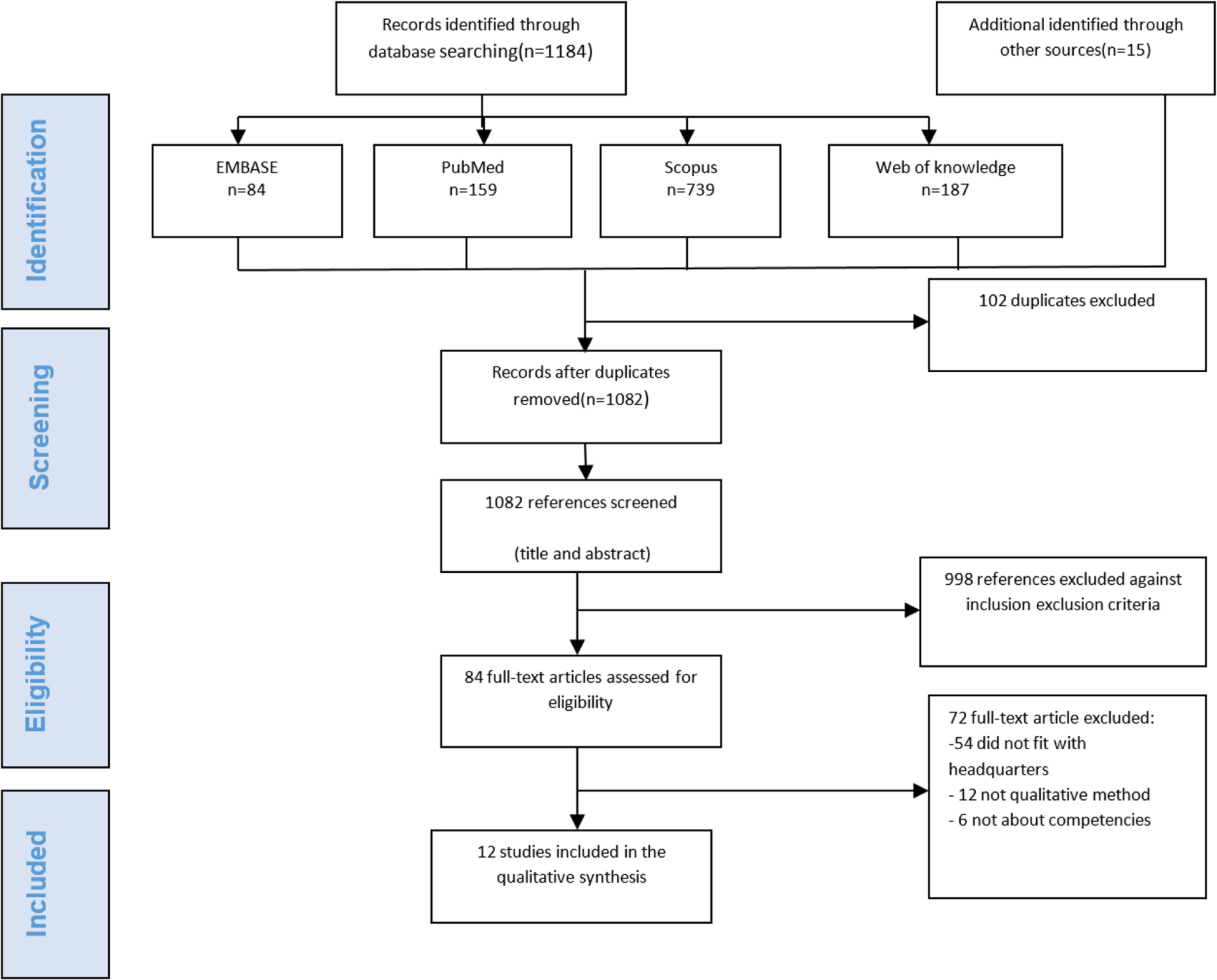
Flow Diagram Showing the Different Phases of Searching for Relevant Publications

In the first stage within the electronic databases, we recognized 1184 publications relevant to Health workers’ competencies. In the last phase, after eliminating the duplicates (*n* = 102), a total of 998 studies were excluded by title and abstract reviewed by two independent authors, leaving 84 studies for full-text review. Complete texts regarding the exclusion and inclusion criteria (i.e., 12 studies from 7 countries) were chosen to include in scoping review. Fig. 1 represents the details related to step-by-step exclusion and inclusion of the works.

### Results of the assessment of the methodological quality

The 12 included papers were reviewed for assessment of methodological quality by all three authors. SRQR items that were poorly reported in qualitative studies included researcher features and reflexivity [26–36], ethics about the human subjects [26–28, 30–32, 34, 35, 37] and techniques to enhance trustworthiness were followed in the corresponding references [27–32, 34–36]. The weakest score was 11 [29] and the best was 20 [37] out of 21. Table 1 provides full details within the scoring process. Overall, within the assessment, a rich source of data was found in the considered literature; however, the nature of weakly scoring items was taken into account over the review procedure comprising potential biases in each work.

**Table 1:** SRQR critical appraisal tool results for qualitative studies

Study	Title &Abstract		Introduction		Methods											Results/findings		Discussion		Others		Score
	Title	Abstract	Problem formation	Purpose or research question	Qualitative approach & research paradigm	Researcher characteristics & reflexivity	Context	Sampling strategy	Ethics pertaining to subjects human	Data collection methods	Collection instruments & tech	Units of study	Data processing	Data analysis	Techniques to enhance trustworthiness	Synthesis & interpretation	Links to empirical data	Integration with prior work, transferability	Limitations	Conflict of interest	Funding	Out of a possible 21
	**S1**	**S2**	**S3**	**S4**	**S5**	**S6**	**S7**	**S8**	**S9**	**S10**	**S11**	**S12**	**S13**	**S14**	**S15**	**S16**	**S17**	**S18**	**S19**	**S20**	**S21**	
**Ablah et al** [33]	X	X	X	X	X		X	X	X	X	X	X	X		X	X	X	X	X			17
**Akbar et al** [28]	X	X	X				X	X		X	X	X	X	X			X	X			X	13
**Bornstein et al** [34]	X	X	X	X	X		X	X		X	X	X	X	X		X	X	X			X	16
**Conejero et al** [35]	X	X	X	X	X		X	X		X	X	X	X	X			X			X	X	15
**Council on Linkages** [27]	X		X	X	X		X			X	X	X					X	X			X	11
**Damari et al** [26]	X	X	X	X	X		X			X	X	X			X	X		X	X			13
**Joqerst et al** [32]	X	X	X	X	X					X	X	X	X	X			X	X	X	X		14
**Lamb et al** [31]	X	X	X	X	X					X	X	X	X	X		X				X		12
**Margaret et al** [30]	X	X	X	X	X		X	X		X	X	X	X	X		X		X	X	X	X	17
**Rodriques et al** [37]	X	X	X	X	X	X	X	X		X	X	X	X	X	X	X	X	X	X	X	X	20
**Whittaker et al** [36]	X	X	X	X	X		X	X	X	X	X	X	X	X			X	X	X	X	X	18
**Wright et al** [29]	X	X	X	X	X		X		X			X				X				X	X	11

### The characteristics of the included studies

The 12 studies were published in the 12 years from 2005 to 2017. These studies were conducted in 6 developed countries (USA, Australia, Canada, Ireland, Spain, and the UK) [27–35, 37] and one study in developing countries (Iran) [26] (Table 2). The focus of this study was on the staff members of public health that work in the health sector. All 12 studies used qualitative designs (Delphi/focus group/Semi-structured/Structured/call interviews or expert panel). Table 2 presents the summary characteristics of the included studies.

**Table 2:** Summary characteristics of the studies (theme reported: I = Leadership and management competencies, II = Analyzing, Interpreting and Reporting competencies III = Public Health knowledge competencies, IV = Interpersonal relationship competencies, V = Personality competencies, VI = Cultural and community competencies, VII = International/ Global health competencies)

Author/year/country	Aim	Study design	Population	Data collection	Data analysis	Theme reported by authors
**Ablah et al (2014) USA** [33]	**To develope standardized global health competency model existed for master-level public health students**	**Qualitative**	**Expert- N:11**	**multistage modified-Delphi process**	**Thematic**	**I, II, III,V, VI**
**Akbar et al(2005) Australia** [28]	**To identify the core competencies they require, and the implications for education and training of international health Practitioners.**	**Qualitative**	**Consultants, key NGOs Members and education and training providers-*N*** **= 19**	**Semi-structured interviews/ group interview**	**Frame work analysis**	**I, II, III, IV, VI**
**Bornstein et al (2015) Canada** [34]	**To develop an enriched set of core competencies for health services and policy research (HSPR) doctoral training**	**Qualitative**	**relevant stakeholders groups-*N*** **= 19**	**Semi-structured interview**	**Triangulation**	**I, II, III, IV, VI**
**Conejero et al(2013) USA** [35]	**To find the core competencies that all professionals working in public health and health service delivery should possess**	**Qualitative**	**Experts-N:225**	**Expert panels**	**Frame work analysis/ situation Analysis**	**I, II, III, IV, VI, VII**
**Council on Linkages (2014) USA** [27]	**To find Core Competencies for Public Health Professionals**	**Qualitative**	**Experts-N: 350**	**Conference call/ Workgroup**	**Not exist**	**I, II, III, IV, V, VI**
**Damari et al (2017) Iran** [26]	**Aims to determine the competencies of the activists in public health.**	**Qualitative**	**Stakeholders/members from faculties-*N*** **= 7**	**Structured interview/Focused group discussions**	**Content analysis method**	**I, II, III, IV, V, VI**
**Joqerst et al (2015) USA** [32]	**To describe the Subcommittee’s work and proposed list of inter professional global health competencies.**	**Qualitative**	**Experts- N:11**	**Call interviews**	**Mixed method**	**I, II, III, IV, V, VI, VII**
**Lamb et al (2010) USA** [31]	**To identify inter professional competencies in healthcare**	**Qualitative**	**Not exist**	**Not exist**	**Narrative**	**I, II, V**
**Margaret et al (2012) Ireland** [30]	**To Developing Competencies and Professional Standards for Health Promotion in Europe**	**Qualitative**	**Stakeholders*N*** **= 33**	**Delphi survey monkey**	**Delphi survey/ Focus groups**	**I, II, IV, V, VI**
**Rodriques et al (2013) Spain** [37]	**To identify current and future competencies (managers and technicians) for public health professionals in Catalonia**	**Qualitative**	**Health professionals*N*** **= 31**	**Semi-structured interview**	**Phenomenological approach**	**I, II, III, IV, V, VI, VII**
**Whittaker et al (2015) Australia** [36]	**To identify the minimum HIS competencies that could be expected in Low and middle income countries that do not have advanced technology**	**Qualitative**	**HIS Specialist**	**Delphi approach, expert panel**	**Content analysis**	**I, IV**
**Wright et al (2008) UK** [29]	**To describe how the UK Public Health Skills and Career Framework was developed**	**Qualitative**	**Experts**	**workshops**	**Not exist**	**I, II, III, IV, V, VI**

### Thematic analysis results

#### The first theme: leadership and management competencies

These competencies help health headquarters to coordinate human and non-human resources for achieving the goals of health care organizations acceptably. The competencies of this domain under the title of overall management and leadership cover the process of planning, organizing, leading, and controlling the collective efforts of the organization’s employees and the optimal use of resources to meet the organizational goals. Thus, the set of competencies in this area helps to coordinate the collective efforts of health headquarters to provide, maintain, and promote the health of the community covered by the effective and efficient use of available resources. Based on the results of this study, this theme consists of the following 14 sub-themes and accounts for about 42% of the total code.

#### Policy development competencies

This sub-theme is the need for health systems for making major policies and the role of health systems in society. In particular, the emphasis on the role of health systems in public health makes inevitability for the importance of staffing in evidence-based policymaking and its implementation in the health system.

Based on the obtained results, headquarters should have policy formulation skills [26, 34] and the ability to identify where new policies and strategies are needed (i.e., identify opportunities for policy development) [29]. They should have the ability to develop and implement policies and strategies in the work arena [29].

“Identify opportunities for policy development that will improve health and wellbeing and reduce inequalities” [29].

Also, the ability to interpret allows applying local, regional, national policies, and strategies in the field of the health sector [27]. In this regard, gathering information that can provide options for policies for headquarters is of great necessity [27]. To better evaluate these policies, they need to have information about policies.

#### Planning competencies

The process planning has distinct and interconnected steps to produce a coherent output in a coordinated system of decisions. Planning is not thinking about the future or controlling it, rather than doing these affairs. Although planning is not a typical manner of decision-making, it allows making a set of coordinated decisions. Therefore, it is a vital skill for health headquarters.

“Recognize that planning and decision-making are social, technical and political processes to guarantee better health outcomes” [35].

Headquarters should have the ability to set strategic direction and vision for health [29]. Then, they should know about planning and program management [37] and the ability to plan and manage health programs [26]. Based on the obtained results, they develop a feasible action plan within the resource [30]. Also, the ability to use community health assessments for improving planning [27], preparedness, and planning for public health emergencies [37] contributes to the development of the organizational strategic plan [27], having ability and knowledge about project management [32, 33], and applying scientific evidence throughout program planning for health development [32, 33].

#### Management control competencies

The concepts of supervision in management or control in management all refer to comparing one thing between what it is and what it should be. This is one of the main principles of management.

“Monitors current and projected trends e.g., health, fiscal, social, political, environmental representing the health of a community” [27].

Anyone with managerial activity should at least be familiar with the basics of control since [26, 35] control is one of the basics and principles of management. The ability to identify and apply important and measurable indicators in systems [27] is one of the most important skills needed by health headquarters.

#### Performance management competencies

Performance management in the organization makes it possible to achieve the goals effectively and permanently and to improve the performance of the employees and the whole organization, as well as increasing the productivity and efficiency of the organization.

“Develops performance management systems e.g., using informatics skills to determine minimum technology requirements and guide system design, identifying and incorporating performance standards and measures, training staff to use system” [27].

The ability of health headquarters to plan tasks and set expectations for continuous monitoring of the performance [27], development and expansion of the performance, periodic performance ratings, and rewarding good performance are among the capabilities required in this category [37]. Also, the ability to develop performance management systems [35] is based on using performance management systems for organizational improvement and program.

#### Financial planning competencies

In comprehensive financial planning, today’s financial status of the organization will be examined and financial goals will be considered in the near and far future. Finally based on these goals, the method(s) suitable for achieving these goals and their availability will be examined.

“ability to absorb and manage financial resources and advocacy from politicians.” [26].

For this subtheme, health headquarters should have the ability to explain healthcare funding mechanisms and procedures [26, 27], describe financial analysis methods in making decisions [27, 35], develop and defend program budgets [27], and manage programs within projected budgets [27, 28]. They should have the ability to ensure programs are managed within the forecasted budget and justify programs for program budgets [27].

#### Implementation competencies

Implementing policies and procedures of the administrative units [37], implementing strategies for continuous quality improvement [27], and capability for managing the resources required for the operative implementation of plans [30] are basically the main competencies that they should have.

“Implement effective and efficient, culturally sensitive, and ethical health promotion action.” [30].

#### Team management and people supervision competencies

Team management is the capability of a person or an organization for administering and coordinating a group of people to conduct a task.

“Engage and lead a group to influence positively the population’s health and wellbeing” [29].

Based on this subtheme, health headquarters should have knowledge in managing their staff [29, 37] and establish teams to achieve organizational objectives [27]. They should have the ability to organize, lead, and support various teams [34]. Other competencies in this part are implementing evidence-based to enhance team performance [31] and the ability to interdisciplinary work in an organized mode [28, 35].

#### Empowerment and consultation competencies

Empowerment is a set of actions planned for increasing the level of self-determination and autonomy in the individuals and in societies for enabling them to provide their interests in an accountable and self-determined manner based on their authority.

“They can empower individuals and organizations involved in health interventions in such way that they can provide best possible leadership. Consultation to organizations or consultation in national and international level are examples of these skills” [26].

Facilitating the development of personal skills [30], describing the needs for professional development [27], defining the ways to enhance the individual and program performance [27], designing sustainable workforce development for resource-limited [33], and motivating staff members to achieve organizational objectives [26, 27] are among the essential competencies for headquarters in this subtheme.

#### Knowledge management competencies

Within this process, the information and knowledge of an organization are created, shared, used and managed. It denotes a multidisciplinary method to achieve the organizational goals by using knowledge in the best way.

“Incorporate new knowledge to improve practice and respond to emerging challenges in health” [30].

Indeed, it is the ability of knowledge management [37] and incorporating new knowledge to respond to challenges in the health sector [30].

#### Change management competencies

This is a collective expression for all methods for preparing, supporting, and helping teams, organizations, and individuals for organizational change.

“Ability to plan, manage, and implement change, including to communicate a clear vision and rationale for change” [34].

Ability to describe the impact of changes in the organizational process [27, 34] and making changes within a politically challenging environment [29] are some competencies in this subtheme.

#### Resources management competencies

This process is the effective and efficient development of the resources of an organization when required.

“Secure, prioritize and allocate resources to achieve optimal impact on population health and wellbeing” [29].

Headquarters should have the ability to explain the ways resources and assets can be utilized to enhance health [27], to gather information resources [26], to contribute to mobilizing and managing resources [30], and secure, prioritize, and allocate resources [29] to achieve optimal impact on population health.

#### Risk management competencies

Risk management is to identify, evaluate, and prioritize the risk.

“Provide an immediate response to threats, risks and damage from disasters based on the risk assessment, in order to protect health” [35].

They should have the ability to identify threats, risks, and damages to health and provide an immediate response to them [35]. Other important competencies are to perform disaster risk assessments, design disaster risk management plans for natural hazards, and plan and implement post-disaster reconstruction [29].

#### Conflict management competencies

Capacity to manage conflicts between people [28, 37], apply strategies to minimize conflicts and Apply strategies to minimize conflicts [31] Are some of the important competencies that health headquarters should have.

“Apply strategies to minimize conflicts in perspective and timing across professions and stakeholders” [28].

#### Information management competencies

Information management includes an organizational action cycle including acquiring information from one or more sources, the custodianship and the distribution of the information when required, and its ultimate disposition by elimination or archiving.

“Manage information, research and other knowledge relevant to daily practice to improve the outcomes of health actions and contribute to the well-being of the population” [35].

Having basic information management competencies (i.e., the ability of data management [29, 36], having knowledge about information systems and technologies [37], ability to use information analytically and critically [37], and search and identify the scientific evidence [37], data generation competencies (i.e., accesses, processes, and data analysis, ensuring the accuracy of data collection, and reports preparation, ability to use ICT infrastructure (i.e., using information technology in accessing, collecting, analyzing [29, 36], and using applications for structured data entry, applying basic computer technology skills, and using available communication infrastructure [27, 36], ability to use data (i.e., making decisions based on surveillance data and effect of policy and priority setting through surveillance data [30, 36] are some of the important competencies in this subtheme.

#### The second theme: analyzing, interpreting and reporting competencies

The analysis is the procedure to break a complex substance or topic into smaller parts to obtain a better comprehend it. Interpretation is the act of explaining, reframing, or otherwise showing your understanding of something.

#### Basic analysis competencies

Knowing the application of research [37], managing research, and other knowledge to enhance the results [35], ability to use and analyze quantitative and qualitative [26, 27, 30, 37], having basic computer skills to work with computer scientists [37], and the ability to retrieve evidence from print and electronic sources [27] are some important basic competencies in this subtheme.

“Develop methodologies, technologies and good practices for the management, analysis and communication of health information” [35].

#### Identifying a topic, problem, or issue

Managers should identify internal and external facilitators and barriers of the health system [27], perform a situation analysis within the health context [32, 33, 37] and finally set public health priorities [26, 29, 30, 32]. Other competencies include the ability to analyze the influences of health system issues on the decision [31], the ability to analyze the distribution of resources to meet the health needs [33], and the ability to conduct routine gap analyses [37].

“Determine priorities for research and development into population health and wellbeing” [29].

#### Gathering information and interpreting

Providing data and evidence for executives and policymakers [26], having competence in applying the concepts and data [31], generating information for evaluating health service performance [34, 37], collecting valid and reliable quantitative and qualitative data [27], interpreting qualitative and quantitative [27], and interpreting public health data to answer questions [29] are some of the important competencies in this sub-theme.

“The ability to collect, analyze, interpret, and use a wide range of data and to reflect critically on and iteratively incorporate theory and research evidence” [34].

#### Developing solutions or furthering your understanding of topics

Based on the result of this study, applying evidence-based approaches to solve issues [26] and set a culture of continuous evidence-based improvement are important competencies for health headquarters [26].

“research process from determining the need to prioritizing the topics and follow up their publication and make sure that the findings has been applied” [26].

#### Testing solutions or new ideas based on what they have learned

applying the results of the research to the population [37]. Indeed, they should have the ability to utilize evidence-based strategies and research [30] and ability such that recognizing limitations of the evidence [27].

“Evaluate the benefits and costs of design solutions” [31].

#### Post-analysis, or reviewing what solutions worked competencies

Having health plans evaluation skills [26], ability to incorporate the assessment into the preparation and implementation [30], ability to utilize assessment results to refine and enhance health promotion action [30], the ability of recommended pro-health actions for various target audiences [37], and analysis and evaluation of health-related policies and programs [34] are needed for any health headquarter.

“Use evaluation findings to refine and improve health promotion action” [30].

#### The third theme: public health knowledge competencies

Public health knowledge is responsible for gathering, analyzing, and interpreting information. This theme contains six subthemes, as follows:

#### Basic public health skills

Having a basic knowledge of service public health to explain using public health sciences in the delivery of health [27, 28, 37] and the ability to define noticeable events in public health history [27] are some of the competencies in this area.

“Describes how public health sciences Public Health Services” [27].

#### Knowledge of vision, mission, goals, and health strategies

Headquarters should know the dominant values in a health system [26] and public health should know the lines of the institution where they work [37] and mission, values​​, and objectives of the Public Health Agency. Also, they should have an integrative vision of the health concept [37].

#### Knowledge of health structures

Knowing the organization in which they work [34, 37], knowledge of the public health organization chart [37], and the ability to describe public health as part of a larger inter-related system [27] are important for health headquarters.

“Describes public health as part of a larger inter-related system of organizations that influence the health of populations at local, national, and global levels” [27].

#### Knowledge of health procedures

All headquarters should have a collective understanding of health procedures related to them [26]. Also, they should have the ability to apply public health science in the field of management programs [35].

“Understand result chains, knowledge of primary health care” [26].

#### Ability to apply public health science in field competencies

Ability to apply public health sciences in the delivery of Public Health Services is a crucial skill that headquarters should have [26, 37].

“Applies public health sciences e.g., biostatistics, epidemiology, environmental health sciences, health services administration, social and behavioral sciences, and public health informatics in the delivery of the Essential Public Health Services” [27].

#### Health equity and social justice

They should have the ability to support the principles of equity [33, 37], implementation plans, activities, and strategies to increase equity [29, 37], encourage quality assurance, and safety standards to decrease inequity [37].

“Apply social justice and human right principles in public health policies and programs” [33].

#### Health promotion and social participation

The managers are required to have the impact on the health of the population [37], develop strategies using the health promotion approach [35], evaluate the concentration and scope of health promotion through required assessments, to obtain positive alterations in community and individual health [35], and the ability to design health communication, media advocacy strategies, and social marketing to enhance health in people and communication [35].

“Facilitate the creation and improvement of participatory social spaces and processes that foster an understanding of health” [35].

#### The fourth theme: interpersonal relationship competencies

The interpersonal competence domain integrates the headquarters’ ability to interact with others and with the greater community.

“Use interpersonal communication and group work skills to facilitate individuals, groups, communities, and organizations to improve health and reduce health inequities” [30].

Having empathy skills (e.g., the ability to use common sense) [30, 34, 37], ability to negotiate (e.g., improving the health through effective use of negotiating) [26, 28, 29, 37], communication skills (e.g., communication through new technologies such as the skill of media utilization) [26, 30, 35].

“Facilitates communication among individuals, groups, and organizations” [27].

and ability to use effective communication skills [26–30, 34, 37] (e.g., communicating in writing and orally, cultural proficiency, the ability to use multiple method of communication, and using culturally appropriate communication methods and technique), Teamwork skills [30, 37] (e.g., the ability to work in multidisciplinary teams, feeling of belonging to the group and the organization, orienting the work toward the success of the team, and using interpersonal group work skills), the ability to work in a network [27, 30, 34–37] (e.g., the ability to manage public health networks, network with motivating stakeholders, contribute to the incorporated care networks with various level, work collaboratively across the sectors to affect public policy, and facilitate the development and sustainability to the network).

#### The fifth theme: personality competencies

Personal competencies are personal abilities and traits affecting the results in life and the workplace.

Subcategories in this part include having ethical reasoning competencies. “Use ethical, empowering, culturally appropriate, and participatory processes to implement health promotion action” [30] (e.g., the ability to apply ethical principles in any public health subject, incorporate ethical standards into all interaction, and the ability to understand and resolve common ethical issues) [26, 27, 30, 32, 33, 37].

Critical thinking skills (e.g., the ability to analyze information objectively and make a reasoned judgment) [37], problem-solving ability (e.g., using methods in an orderly manner to find solutions to problems) [30, 31, 37], attitude toward public health, self-awareness skills (e.g., the ability to be aware of or to recognize the emotions, behaviors, beliefs, and motivations) [27, 37], deciding skills (i.e., a cognitive procedure leading to selecting a belief or a course of action among numerous alternative possibilities) [26, 27, 29, 30, 37].

“Makes evidence-based decisions e.g., determining research agendas, using recommendations from The Guide to Community Preventive Services in planning population health services” [27].

Creative thinking skills (e.g., developing an idea from the beginning to the end and the ability to work in a systematic and structured and organized way) [31, 37], individual updating skills (e.g., staying updated on knowledge, being completely aware of global reports related to the health sector, being informed of the latest scientific articles, and having the ability to adapt skills) [26, 32, 37].

#### The sixth theme: cultural and community competencies

Cultural competence is the capability of participating effectively and ethically in professional and personal intercultural settings. This them demands being aware of one’s own cultural values and worldview and their implications for making reflective, having reasonable and respectful choices, and the capability in imagining and collaborating across cultural boundaries.

#### Understand the role of community health actors

This subtheme is containing the ability to Describes agencies with authority to impact the community health [27], communicate joint lessons learned to community partners, show diplomacy, build trust with community partners [32], and explain the programs and services presented by governmental and non-governmental sectors [27].

“Describes government agencies with authority to impact the health of a community” [27].

#### Strategies and activities for diversity in the community

Diversity affects policies and programs [27]. The concept of diversity is among the important competencies in this field [28].

“Recognizes the ways diversity influences policies, programs, services, and the health of a community” [27].

#### Socio-cultural and political awareness

This subtheme includes showing commitment to social responsibility [32], evaluating the impacts of programs, services, and policies on various populations in a community [27, 33], and the ability to list major economic and social factors of health and their influences on the quality and access to health services [32].

“Assesses the effects of policies, programs, and services on different populations in a community” [27].

#### Community strengthening

This subtheme includes engagement of community members to enhance health in a community [27], integrate community assets and resources to improve health [32], develop strategies that strengthen community [33], and inform the public regarding programs, resources, and policies [27], and improving the health in society [33].

“Cocreate strategies with the community to strengthen community capabilities, and contribute to reduction in health disparities and improvement of community health” [32].

#### Advocacy skill

collaborating, partnering competencies [26, 28], developing procedures for managing health partnerships [33], working with stakeholders to agree on a shared vision [30], using diplomacy with partners [33], engaging with and influence key stakeholders [30], and employing participatory methods to engage stakeholders and advocate across sectors [30].

“To be able to convince the authorities in three powers of executive, legislative, and judiciary, and even higher than that in regional and international levels” [26].

#### Building relationships with people from different cultures

This subtheme includes engagement with community members to enhance health in a community [27], create relationships to increase health in a community [27], recommend some relationships to enhance health [27], communicate effectively with people from a different culture [28], addressing the needs of diverse populations in the culture they work [27], and working independently in a complex cultural setting [27].

“Establishes relationships to improve health in a community” [27].

#### Understanding of culture competencies

Knowing sociology is one of the important skills that health headquarters should have [26–28, 32]**.**

#### The seventh theme: international/ global health competencies

The knowledge generation and its optimal use and application, particularly in the field of development and global health, have attracted considerable attention recently.

Based on the results of this study, three subthemes emerged in this part are global health knowledge (e.g., a basic understanding of the relationships between health, global inequities, and human rights and identifying the global dimensions of local health action) [32, 35].

“Describe different national models or health systems for provision of health care and their respective effects on health and health care expenditure” [32].

Global health vision (e.g., global and integrative vision of public health) [32, 37].

“Recognize the local implications of global health events to understand global interconnectivity and its impact on health conditions in the population” [35].

, and absorb global advocacy for health (e.g., collaborating with diverse communities to solve health problems, engaging in international non-governmental organizations, and encouraging the advocacy) [32, 35].

“Contribute effectively to the care of vulnerable groups, especially migrants, travelers, transnational ethnic minorities and border populations, for the mitigation, eradication and/or control of global health problems” [35].

Table 3 shows the themes, subthemes, and codes of selected studies.

**Table 3:** Themes, subthemes and codes of selected studies

Themes	Subthemes	Example of codes	Sub-themes References
Leadership and management competencies	**Policy Development competencies**	- **Identify where new policies and strategies are needed** - **Develop and implement policies and strategies in area of work** - **Examines the feasibility and implication of policies**	[26–29, 34, 35]
	**Planning competencies**	- **Set strategic direction and vision for health** - **Contributes to development of organizational strategic plan** - **Knowledge about planning and program management**	[26–33, 35]
	**Management control competencies**	- **Lead on the evaluation of interventions** - **control the organization** - **Monitors current and projected trends**	[26–29, 35]
	**performance management competencies**	- **Develops performance management systems** - **Uses performance management systems for program** - **Describes program performance standards and measures**	[27, 35]
	**Financial Planning competencies**	- **Uses administrative processes for budgeting** - **Develops program budgets** - **Defends program budgets**	[26–28, 35, 36]
	**Implementation competencies**	- **Implement interventions** - **Implements policies and procedures of the administrative units** - **Implements organizational strategic plan**	[27, 30, 37]
	**Team management and people supervision competencies**	- **Apply leadership practices that support team effectiveness** - **Lead interdisciplinary groups that work in a coordinated manner** - **Describes how teams help achieve program, organizational goals**	[26–32, 34, 35, 37]
	**Empowerment and consultation skill competencies**	- **Facilitate the development of personal skills** - **Ensures use of professional development opportunities** - **Describes ways to improve individual and program performance**	[26, 27, 30, 32, 33]
	**Knowledge management competencies**	- **Apply technical knowledge in work** - **Knowledge management capacity** - **Incorporate new knowledge to respond challenges**	[30, 37]
	**Change management competencies**	- **Anticipating the changes in your environment** - **Managing change in response to dynamic, evolving** - **Describes the impact of changes on organizational practices**	[27, 30, 34, 36, 38]
	**Resources management competencies**	- **Describes and identifies assets and resources** - **Select credible sources** - **Contribute to mobilizing and managing resources**	[26–31, 35]
	**Risk management competencies**	- **Perform disaster risk assessments** - **Design disaster risk management plans for natural** - **Design investment projects for reducing the health risks**	[29, 35]
	**Conflict Management competencies**	- **Apply strategies to minimize conflicts** - **Apply strategies to minimize conflicts** - **Capacity to manage conflicts between people**	[28, 30, 31, 37]
	**Information management competencies**	- **Knowledge about information systems** - **Knowledge about information technologies** - **Ability to analyze information to know the health status**	[27, 29, 30, 35–37]
Analyzing, Interpreting and Reporting competencies	**Basic analysis competencies**	- **Basic computer skills to work with computer scientists** - **Describes factors affecting the health** - **Analyzes quantitative and qualitative**	[26, 27, 29, 32, 35, 37]
	**Identifying a topic, problem or issue competencies**	- **Analyze distribution of resources to meet the health needs** - **Determine priorities for research** - **Assess the needs and problems of individuals, families, groups**	[26, 27, 29–33, 35]
	**Gathering information and Interpreting competencies**	- **prioritize, interpret** - **providing data and evidence for executives and policy makers** - **Accurately interpret the dominant**	[26, 27, 29–31, 33–35, 37]
	**Developing solutions or furthering your understanding of topics competencies**	- **Set a culture of continuous evidence-based improvement** - **Uses evidence in developing, implementing, evaluating** - **Applying evidence-based approaches to solve issues**	[26, 27, 29]
	**Testing solutions or new ideas based on what you’ve learned competencies**	- **Recognizes limitations of evidence** - **Use culturally and ethically appropriate assessment approach** - **Apply the results of the research to the population**	[27, 30, 31, 37]
	**Post-analysis, or reviewing what solutions worked competencies**	- **Integrate evaluation into the planning and implementation** - **Use evaluation findings to refine and improve** - **Analysis and Evaluation of Health-Related Policies and Programs**	[26, 30, 31, 34, 35]
Public Health knowledge competencies	**Basic public health competencies**	- **Describes prominent events in the history of public health** - **Discusses the scientific foundation of the field of public health** - **Basic knowledge of service quality management**	[27, 28, 37]
	**knowledge of vision, mission, goals and health strategies competencies**	- **Know the lines of the institution where you work** - **Integrative vision of the health concept** - **mission, values ​​and objectives of the Public Health Agency**	[26, 37]
	**Knowledge of health structures competencies**	- **Describes public health as part of a larger inter-related system** - **Global knowledge about the organization in which they work** - **Knowledge of the public health organization chart**	[27, 34, 37]
	**Knowledge of health procedures competencies**	- **Identify minimum or basic safety conditions in health care delivery** - **understand result chains, knowledge of primary health care**	[26, 35]
	**Ability to apply public health science in field competencies**	- **Orientation to the preservation of the health of the population** - **Health protection operations** - **to**	[26, 37]
	**Health equity and social justice competencies**	- **Promote quality assurance, safety standards to reduce inequity** - **Support the principles of equity, using the social determinants** - **Implement plans, strategies and activities that increase equity**	[29, 32, 33, 35]
	**Health promotion and social participation competencies**	- **Knowledge about health promotion and health education** - **Develop strategies using the health promotion approach** - **Assess the focus and scope of health promotion through needs**	[35, 37]
Interpersonal relationship competencies	**Empathy competencies**	- **Empathy, understanding and respect** - **Ability to use common sense**	[30, 34, 37]
	**Negotiation competencies**	- **Improve the health through effective use of negotiating** - **Ability to relate** - **Negotiation capacity (especially in management positions)**	[26, 28, 29, 37]
	**Communication competencies**	- **Communication through new technologies** - **Use effective communication skills** - **Communicate with intelligible way**	[26–30, 32, 34, 35, 37]
	**Teamwork competencies (including interdisciplinary teams)**	- **Work in multidisciplinary teams** - **Feeling of belonging to the group and the organization** - **Team culture (orient the work to the success of the team)**	[30, 37]
	**Networking competencies**	- **Participate in integrated care networks with the different level** - **Interest Facilitate the development and sustainability to network in promoting agreement, consensus and cohesion among** - **Ability to work in a network**	[27, 30, 34–37]
Personality competencies	**Ethical reasoning competencies**	- **Applies ethical principles in any public health subject** - **Incorporates ethical standards into all interaction** - **ability to understand and resolve common ethical issues**	[26, 27, 30, 32, 33, 37]
	**Critical thinking competencies**	- **Have initiative, personal disposition**	[37]
	**Problem solving competencies**	- **problem solving** - **problem solving skills**	[30, 31, 37]
	**Attitude toward public health competencies**	- **Adheres to organizational policies and procedures** - **Rigorous attitude towards everyday tasks** - **Vocational attitude towards public health**	[27, 37]
	**Self-awareness competencies**	- **Work capacity and dedication** - **Involvement and motivation** - **Responsible attitude, adaptability and flexibility**	[37]
	**Decision making competencies**	- **Makes evidence-based decisions** - **Take decisions** - **Willingness to provide decisions**	[26, 27, 29, 30, 37]
	**Creative thinking competencies**	- **Develop an idea from the beginning to the end** - **Ability to work in a systematic, structured and organized way** - **Ability to innovate**	[31, 37]
	**Individual updating competencies**	- **work with a multidisciplinary perspective** - **Stay updated on knowledge** - **Open attitude towards learning**	[26, 32, 37]
Cultural and community competencies	**Understand the role of community health actors competencies**	- **Communicate joint lessons learned to community partners and global** - **Demonstrate diplomacy and build trust with community partners** - **Identifies agencies with authority to address specific health need**	[27, 32]
	**Strategies and Activities for diversity in community competencies**	- **Describes diversity of individuals, populations in a community** - **Describes the concept of diversity as it applies to population** - **Advocates for a diverse public health workforce**	[27, 28]
	**Socio cultural and political awareness competencies**	- **Demonstrate a commitment to social responsibility** - **Assesses effects of policies, programs, services on populations** - **List major social and economic determinants of health**	[27, 32, 33]
	**Community strengthening competencies**	- **Engages community members to improve health in a community** - **Integrate community assets and resources to improve the health** - **Create strategies with the community to strengthen community**	[27, 30, 32, 33]
	**Advocacy skill, collaborating and partnering competencies**	- **Advocates for policies, programs, and resources that improve health** - **Advocate across sectors for the development** - **Ability to absorb advocacy from politicians**	[26–30, 32–35]
	**Building Relationships with People from Different Cultures competencies**	- **Engages community members to improve health in a community** - **Identifies relationships that are affecting health in a community** - **Establishes relationships to improve health in a community**	[27, 28]
	**Understanding of Culture competencies**	- **Work independently in complex cultural setting** - **Adapt to diverse educational and cultural backgrounds** - **Having knowledge of sociology**	[26–28, 32]
International/ Global health competencies	**Global health knowledge competencies**	- **Recognize the local implications of global health events** - **Recognize the global dimensions of local health action** - **Describe general trends& influences the global availability of HRH**	[32, 35]
	**Global health vision competencies**	- **Apply social justice and human rights principles in addressing** - **Ability of global vision** - **Global and integrative vision of public health**	[32, 37]
	**Absorb global advocacy for health competencies**	- **Engage non-governmental organizations and encourage advocacy** - **Collaborating with diverse communities to solve health problems**	[32, 35]

## Discussion

To increase its governance capacity and the efficiency and effectiveness of the health headquarters, every country needs manpower delivering better services to citizens based on the public interest and improving their capabilities and competencies following the increasing growth of changes [38, 39]. Although the process of designing the subject of competency management emerged and appeared less frequently in the public and governmental sectors, some scholars considered it as a lever for government agencies to turn existing bureaucracies into efficient and flexible units [40].

The technical capacity of health care Health headquarters is required to transform policies and decision making into effective executive processes. As much as the health system needs professionals and clinical staff, it requires Health headquarters with the ability to make policy, plan, and use evidence and data to adopt appropriate procedures to achieve their goals.

Our results showed that the focus of the studies was on management and leadership competencies in health headquarters. Owing to the numerous challenges in the field of health, the set of these competencies is particularly important. The health-related activities have a group working nature; indeed, a set of resources work together in harmony to achieve the objectives. Moreover, as healthcare headquarters play a major role in supervising and directing the workforce and improving the efficiency and effectiveness of health organizations activities, they must have relevant skills. This set of skills is not only emphasized in health care organizations but in other organizations is concentrated as the basic competencies [41–43].

The emergence of more technologies is a potential factor in growing information resources [44]. If the generated data cannot be analyzed, producing all this data is not valuable at all [45]. Therefore, the competencies related to analysis, interpretation, and reporting are used to analyze the large or small data, simple or complex, structured or unstructured, quantitative or qualitative data for specific purposes of understanding, forecasting, and optimization. The combination of these competencies creates a product that is used as an input for the decision-making process [46]. In other words, the analysis process creates a large or small data product providing input to another process [47].

Besides, existing basic knowledge of public health is crucial for a better understanding of the conditions governing this system. In fact, health headquarters must be familiar with the goals and strategies, structures, and practices to help the health system achieve its goals by integrating this knowledge with other skills [48].

The fourth theme of competencies relates to interpersonal skills. Indeed, health organizations need to function properly to establish effective relationships with community members and employ social interactions to improve health indicators and related outcomes. This set of indicators helps health care staff to move toward meeting their health needs while creating a common sense with the community.

Another theme of the required competencies is personal skills. These skills are not inherent and thus they can be acquired and expanded. The personal skills can be learned just as easily as other skills. Learning different skills in every area not only helps people in their personal lives but also helps them succeed in their careers. Therefore, it is important to consider upgrading these skills to improve and optimize performance.

Another theme of competencies is related to culture and society. Since culture affects numerous aspects of human life, it also plays a key role in shaping the behaviors, beliefs, and values of one’s health. Hence, health headquarters must be able to communicate effectively with different cultures to be aware of their community needs [48]. Cultural competencies are comprehending society’s values, beliefs, and practices. According to the results of other studies, cultural competencies are an important issue in the activities of health headquarters [49].

Ultimately, the health system of a country or region cannot be regarded as a separate and unaffected part of the global health system. Therefore, health headquarters should include a set of knowledge, skills, and abilities to work in such a system. Indeed, they must have a broad view of global health and the factors involved [46].

### Limitations

One of the main limitations of this study was the small number of articles available on the competencies of health headquarters. The research team attempted to overcome this limitation by using articles available and the level of competencies that is necessary for health headquarters. Another limitation was that most studies (11 cases) were conducted in developed countries. Given the prevailing conditions and facilities in other countries, it may be difficult to apply some of the competencies of this study.

## Conclusion

It is inevitable to list the required competencies of the Health headquarters following the new conditions and the native and cultural requirements and to design the competency model. If an organization employs a well-designed and implemented competency model, it can achieve the best individuals and improve their performance and ultimately business consequences. Based on the results of this study, it is crucial to consider the set of competencies required by the health headquarters when recruiting and employing them, as well as developing training programs to enhance the skills needed to meet the objective.

## Data Availability

All the data analyzed and reported in this paper were from published literature, which is already in the public domain.

## Abbreviations

HR: Human resource
PRISMA: Preferred Reporting Items for Systematic Reviews and Meta-Analyses
SRQR: Critical appraisal tool results for qualitative studies
